# Astrocytes produce nitric oxide via nitrite reduction in mitochondria to regulate cerebral blood flow during brain hypoxia

**DOI:** 10.1016/j.celrep.2023.113514

**Published:** 2023-11-30

**Authors:** Isabel N. Christie, Shefeeq M. Theparambil, Alice Braga, Maxim Doronin, Patrick S. Hosford, Alexey Brazhe, Alexander Mascarenhas, Shereen Nizari, Anna Hadjihambi, Jack A. Wells, Adrian Hobbs, Alexey Semyanov, Andrey Y. Abramov, Plamena R. Angelova, Alexander V. Gourine

**Affiliations:** 1 Centre for Cardiovascular and Metabolic Neuroscience, Department of Neuroscience, Physiology & Pharmacology, University College London, London, WC1E 6BT, UK; 2 College of Medicine, Jiaxing University, Jiaxing, 314001, China; 3 Department of Molecular Neurobiology, Institute of Bioorganic Chemistry, Moscow, 117997, Russian Federation; 4 Faculty of Biology, Lomonosov Moscow State University, Moscow, 119234, Russian Federation; 5 Centre for Advanced Biomedical Imaging, Division of Medicine, University College London, London, WC1E 6BT, UK; 6 The Roger Williams Institute of Hepatology, Foundation for Liver Research, and Faculty of Life Sciences and Medicine, King’s College London, London, SE5 9NT, UK; 7 William Harvey Research Institute, Barts and The London School of Medicine, Queen Mary University of London, London, EC1M 6BQ, UK; 8 Department of Clinical and Movement Neurosciences, Queen Square Institute of Neurology, University College London, London, WC1N 3BG, UK

## Abstract

During hypoxia, increases in cerebral blood flow maintain brain oxygen delivery. Here we describe a mechanism of brain oxygen sensing which mediates the dilation of intraparenchymal cerebral blood vessels in response to reductions in oxygen supply. *In vitro* and *in vivo* experiments conducted in rodent models show that during hypoxia cortical astrocytes produce the potent vasodilator nitric oxide (NO) via nitrite reduction in mitochondria. Inhibition of mitochondrial respiration mimics, but also occludes the effect of hypoxia on NO production in astrocytes. Astrocytes display high expression of the molybdenum cofactor-containing mitochondrial enzyme sulfite oxidase which can catalyze nitrite reduction in hypoxia. Replacement of molybdenum with tungsten or knockdown of sulfite oxidase expression in astrocytes blocks hypoxia-induced NO production by these glial cells and reduces the cerebrovascular response to hypoxia. These data identify astrocyte mitochondria as brain oxygen sensors that regulate cerebral blood flow during hypoxia via release of nitric oxide.

## Introduction

The brain is extremely vulnerable to reductions in oxygen supply (hypoxia) due to its exceptionally high metabolic rate associated with the activities of billions of neurons^1^. If brain blood supply were suddenly to cease, the cerebral oxygen content (~0.03 mM) would be enough to maintain neuronal function for only a few seconds^2,3^. Specialized peripheral (arterial) oxygen sensors are located outside the CNS (in the carotid and aortic bodies) and cannot directly detect brain tissue hypoxia, let alone regional differences in brain tissue oxygenation. This points to the necessity and importance of intrinsic brain mechanism that can sense oxygen^4^ to regulate brain tissue perfusion according to regional differences in oxygen supply/demand, and also increase global cerebral blood flow during systemic hypoxia.

The cerebral vasculature is indeed sensitive to arterial hypoxemia and/or brain tissue hypoxia^5,6^. A number of studies have addressed the potential roles of ATP-sensitive K^+^ channels, as well as signalling mediated by protons, lactate, ATP, adenosine and nitric oxide (NO) in the mechanisms responsible for the dilation of cerebral blood vessels in response to hypoxia^5,6^. However, there are controversies surrounding the functional significance of previously proposed signalling pathways. Understanding these mechanisms is important, as there are significant gradients in brain tissue partial pressure of oxygen (PO_2_) even at normal arterial PO_2_ and saturation^7,8^, suggesting that some brain regions may experience critical reductions of oxygen supply^9,10^. The risk of brain hypoxia increases with aging and in some specific conditions such as exposure to high altitude, lung disease or sleep apnoea.

All penetrating and intraparenchymal cerebral blood vessels are wrapped by the end-feet of astrocytes - omnipresent multifunctional glial cells that control the cerebral vasculature via Ca^2+^-dependent release of vasoactive signalling molecules^11,12^. In this study we tested the hypothesis that astrocytes are brain oxygen sensors which induce dilation of intraparenchymal cerebral blood vessels and increase brain tissue perfusion in response to hypoxia.

## Results

**Figure 1:**
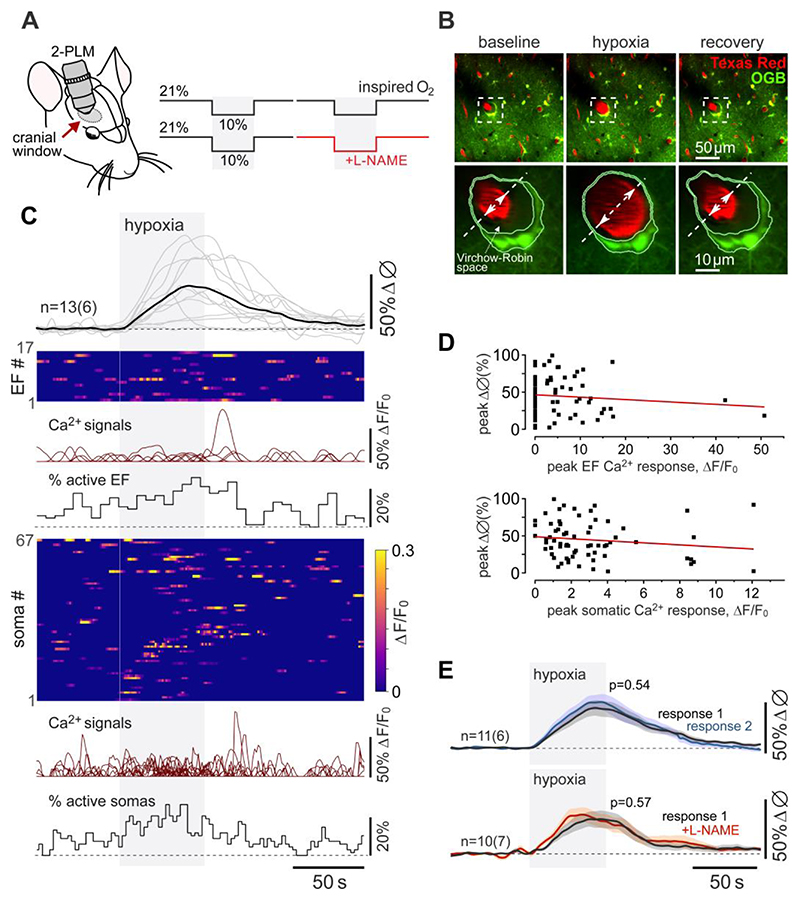
Hypoxia-induced vascular responses in the cerebral cortex. (A) Diagram of the experimental protocol in anesthetized rats instrumented for two-photon imaging of cortical blood vessels (visualized with intravascular dye Texas Red) and Ca^2+^ in cortical astrocytes (using a Ca^2+^-sensitive dye Oregon Green BAPTA 1 AM; OGB). (B) Representative images taken near the cortical surface at baseline, at the peak of the response induced by 10% inspired O_2_, and after a complete recovery from hypoxia, illustrating changes in the vessel diameter and segmentation of the astrocyte endfoot. (C) Hypoxia-induced changes in the diameter of penetrating cortical arterioles (13 vessels recorded in 6 animals), Ca^2+^ signals in perivascular endfeet (EF; 17 regions of interest; ROIs) and cell bodies (67 ROIs) of cortical astrocytes. Ca^2+^ responses in the astrocyte endfeet and cell bodies are illustrated as raster plots, overlays of individual Ca^2+^ traces, and time-binned percentages of active ROIs. (D) Scatterplots showing the relations between the hypoxia-induced Ca^2+^ responses in astrocyte endfeet and cell bodies and the corresponding cerebrovascular responses. (E) Summary data showing overlaid profiles of changes in the diameter of penetrating cortical arterioles in response to two sequential episodes of hypoxia (10% inspired O_2_). Hypoxia triggered reproducible dilations of cortical vessels that were not affected in conditions of established systemic nitric oxide synthase (NOS) blockade with N(ω)-nitro-L-arginine methyl ester (L-NAME; 10 mg kg^-1^). The data are shown as means ± SEM. *n=* number of vessels (number of animals). *p* value, Wald test on linear mixed effect model with random intercepts.

We first investigated whether hypoxia-induced Ca^2+^ responses in astrocytes^13^ might be responsible for the dilations of cerebral arterioles associated with these glial cells. Using 2-photon imaging in anesthetized and artificially ventilated rats, we recorded robust dilations of cortical arterioles (on average by 36±3%; 65 vessels recorded in 23 animals) when the concentration of oxygen in the inspired air was lowered to 10% (Figure 1A-C). Reproducible dilations of cortical vessels occurred immediately after the onset of the hypoxic stimulus (Figure 1B,C). Cortical astrocytes loaded with the Ca^2+^-sensitive dye Oregon Green BAPTA 1 AM responded to systemic hypoxia with increased frequency of Ca^2+^ signals in perivascular endfeet and cell bodies (Figure 1C; Figure S1). It was found that 60% of perivascular endfeet and 55% of astrocyte cell bodies responded to hypoxia with Ca^2+^ signals. However, there were no correlations between the vessel dilations and Ca^2+^ responses in astrocyte cell bodies and endfeet (Figure 1D), suggesting that Ca^2+^-dependent release of vasoactive signalling molecules by astrocytes is unlikely to mediate the cerebrovascular response to hypoxia.

There is evidence that hypoxia-induced increases in cerebral blood flow are mediated by nitric oxide^6,14^. We found that in our experimental conditions hypoxia-induced dilations of cortical arterioles in response to 10% inspired O_2_ were unaffected by systemic nitric oxide synthase (NOS) blockade with N(ω)-nitro-L-arginine methyl ester (L-NAME) (Figure 1E; Figure S2), which is consistent with the results of some human^15^ and experimental animal studies^16^. However, blockade of cGMP signalling was reported to inhibit the cerebrovascular response to hypoxia^17^ and NO can also be produced by reduction of the nitrite anion (NO_2_^-^), - an alternative mechanism of NO generation by certain haem- or molybdenum cofactor-containing metalloproteins that can transfer an electron and facilitate proton donation^18–20^.

**Figure 2:**
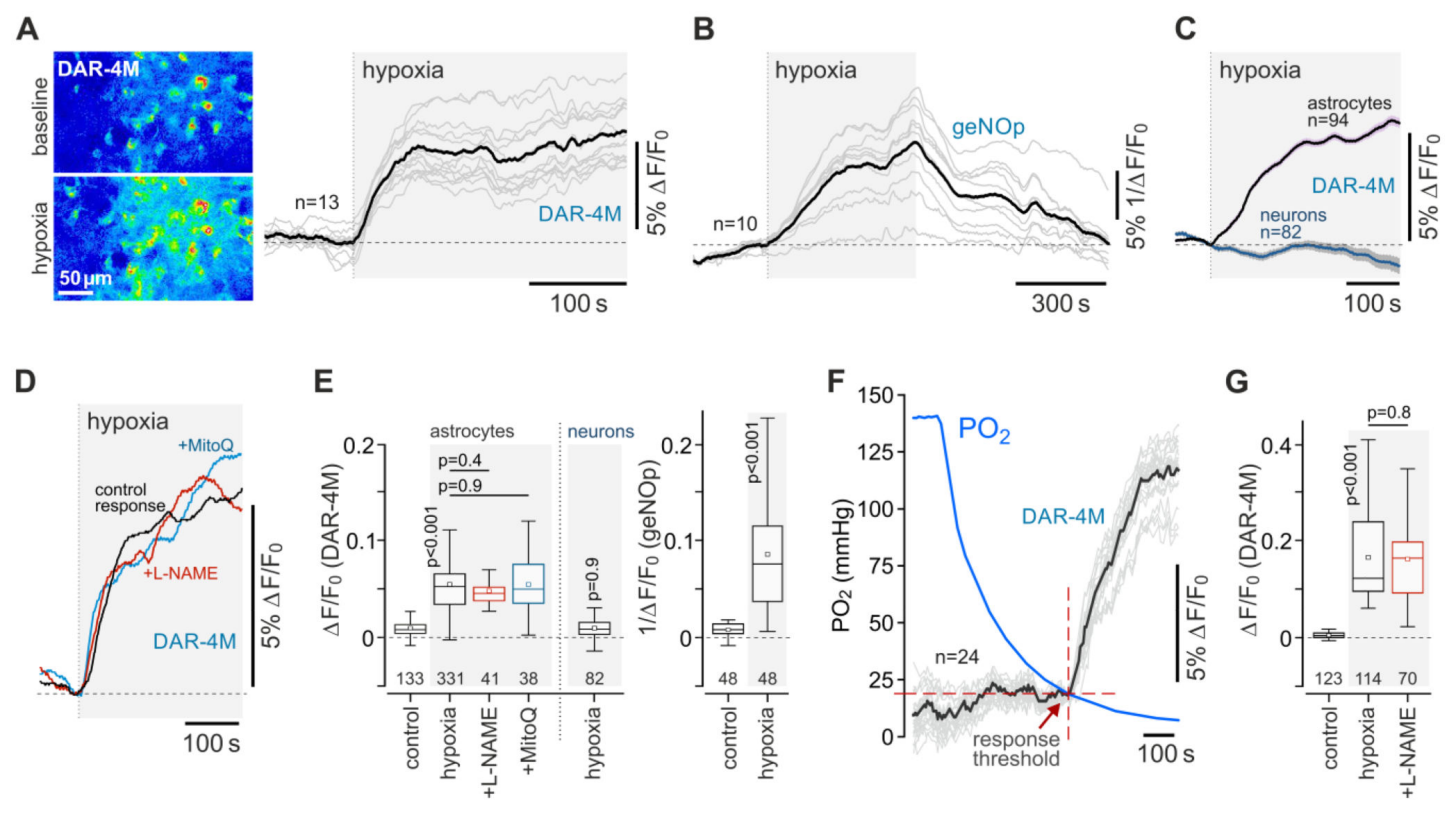
Astrocytes produce nitric oxide in response to hypoxia. (A) Hypoxia-induced changes in fluorescence of a nitric oxide (NO)-sensitive dye DAR-4M in cultured astrocytes. Representative images were taken at baseline and at the peak of the response to hypoxia, induced by the displacement of oxygen by argon. Traces illustrate representative examples of individual (grey) and averaged (black) changes in DAR-4M fluorescence recorded in 13 astrocytes in one experiment. (B) Hypoxia-induced changes in fluorescence of a genetically-encoded NO sensor geNOp expressed in cultured astrocytes. Traces depict individual (grey) and averaged (black) changes in geNOp fluorescence recorded in 10 astrocytes in one experiment. (C) Averaged profiles of hypoxia-induced changes in NO production recorded in cortical astrocytes and cerebellar granule neurons. (D) Hypoxia-induced NO production in astrocytes in the absence and presence of NOS inhibitor L-NAME (100 µM) or ROS scavenger MitoQ (500 µM). Each trace illustrates averaged changes in DAR-4M fluorescence recorded in 8-15 individual astrocytes in separate experiments. (E) Summary data illustrating peak changes in DAR-4M and geNOp fluorescence (reporting NO production) recorded in cortical astrocytes in response to hypoxia, also showing the effects of L-NAME and MitoQ on astrocyte responses, and peak hypoxia-induced neuronal responses. (F) Representative example of data obtained in the experiments in organotypic slices of the cerebral cortex involving simultaneous recordings of NO production by astrocytes and partial pressure of oxygen (PO_2_) at the surface of the slice, illustrating the PO_2_ threshold of the astrocyte response to hypoxia. (G) Summary data illustrating peak hypoxia-induced changes in DAR-4M fluorescence recorded in organotypic cortical slices in the absence and presence of L-NAME. In the box-and-whisker plots, numbers above the horizontal line indicate the numbers of individual astrocytes recorded in 4-8 separate cultures prepared from 3-6 animals. *p* values, ANOVA.

Astrocytes have a loosely assembled mitochondrial respiratory chain, resulting in less efficient mitochondrial respiration, and a propensity to electron leak^21^. We next tested the hypothesis that in hypoxia the electrons from the electron transport chain in astrocyte mitochondria can be used to generate NO via reduction of NO_2_^-^. We used the fluorescent NO indicator DAR-4AM^22^ and the genetically encoded NO sensor geNOp^23^ to record NO production in cultured astrocytes and neurons (Figure 2). Both indicators reported robust hypoxia-induced increases in NO production by astrocytes in response to displacement of oxygen by argon in the incubation medium (Figure 2A-C,E). Hypoxia-induced NO production in astrocytes was unaffected by NOS inhibition with L-NAME (100 µM) (Figure 2D,E,G), or in the presence of a mitochondrial antioxidant MitoQ (500 µM), which scavenges reactive oxygen species, but not NO (Figure 2D,E). Hypoxia had no effect on NO production in cerebellar neurons (known to express high levels of neuronal NOS) (Figure 2C,E). Experiments conducted in organotypic cortical slices, involving monitoring of PO_2_ in the medium, showed that astrocytes begin to produce NO when PO_2_ falls below the threshold of 17±2 mmHg (as recorded on the surface of the slice) (Figure 2F).

**Figure 3:**
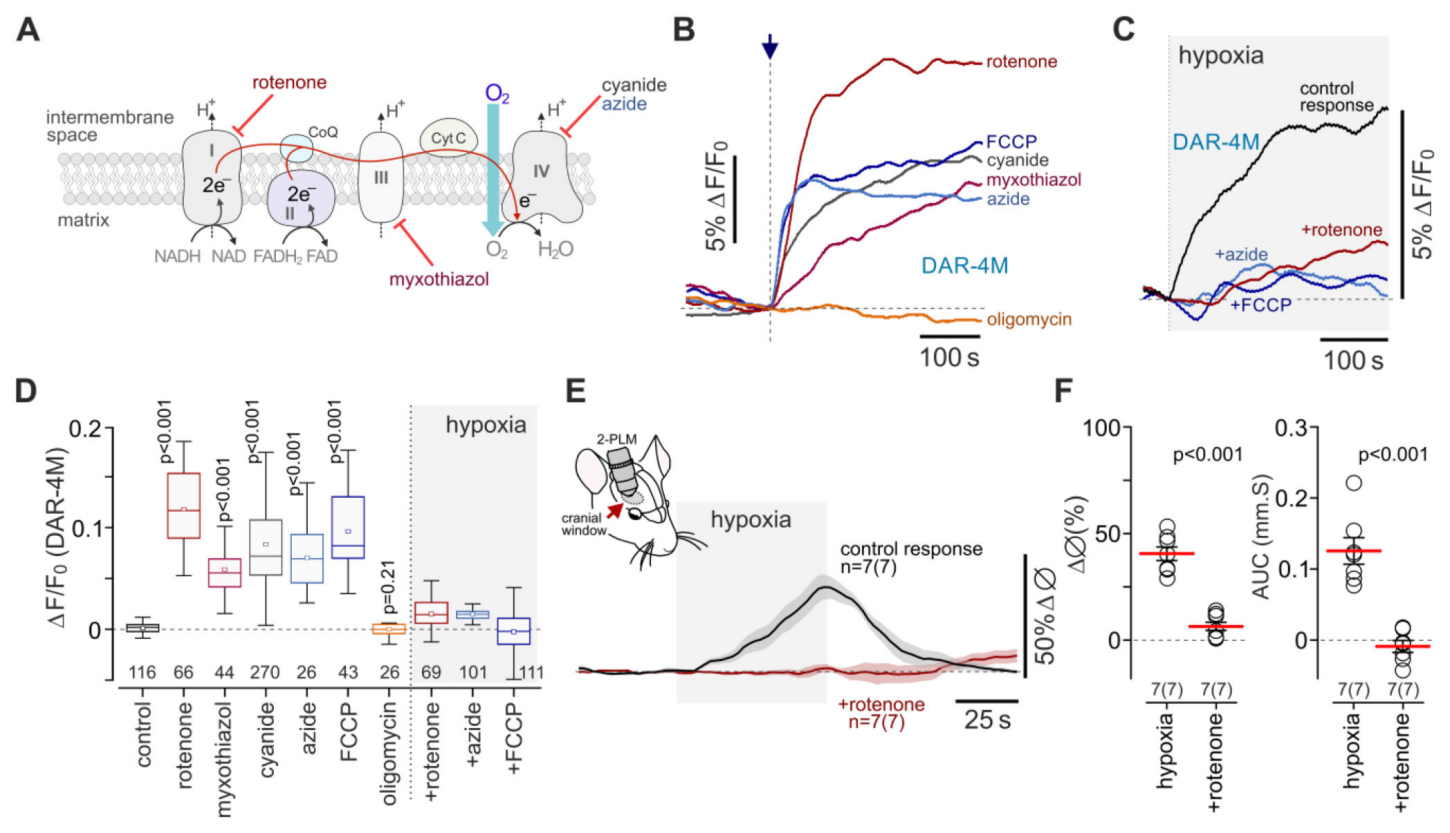
Inhibition of mitochondrial respiration induces nitric oxide production in astrocytes and occludes the responses to hypoxia. (A) Schematic drawing of the mitochondrial electron transport chain (ETC) illustrating the sites of action of the inhibitors used in this study. (B) Inhibition of the mitochondrial ETC or mitochondrial uncoupling (rotenone, 2 µM; myxothiazol, 3 µM; KCN, 100 µM; azide, 0.5 mM; FCCP, 1 µM; oligomycin, 2 µM) trigger NO production in astrocytes. (C) Inhibition of the mitochondrial ETC (rotenone, azide) or mitochondrial uncoupling (FCCP) occlude the effect of hypoxia on NO production in astrocytes. In B and C each trace shows averaged changes in DAR-4M fluorescence recorded in 10-15 individual astrocytes in separate experiments. (D) Summary data illustrating peak changes in DAR-4M fluorescence recorded in cortical astrocytes in response to inhibition of the mitochondrial ETC or mitochondrial uncoupling, and in response to hypoxia in conditions of ETC inhibition or mitochondrial uncoupling. Numbers above the horizontal line indicate the numbers of individual astrocytes recorded in 4-8 separate cultures prepared from 3-6 animals. *p* values, ANOVA. (E) Hypoxia-induced dilations of cortical arterioles are blocked by rotenone applied by microinjection in the vicinity of the recorded vessels (anaesthetized mice). (F) Summary data illustrating peak and integral (area under the curve, AUC) hypoxia-induced changes in cortical arteriole diameter following intraparenchymal microinjections of a vehicle (14% DMSO in aCSF) or rotenone (1 mM; 1.5 µl). The data are shown as individual values and means ± SEM. *n=* number of vessels (number of animals). *p* values, unpaired *t*-test.

It was next found that the effect of hypoxia on NO production in astrocytes could be mimicked by chemical inhibition of the mitochondrial electron transport chain (chemical hypoxia) (Figure 3A). Inhibition of mitochondrial complex I with rotenone (2 µM), inhibition of complex III with myxothiazol (3 µM), inhibition of complex IV with cyanide (KCN, 100 µM) or azide (0.5 mM) or the mitochondrial uncoupling with FCCP (1 µM) triggered robust NO generation by astrocytes and occluded the effect of hypoxia on NO production in these cells (Figure 3B-D). Blockade of complex V with oligomycin (2 µM) had no effect on NO production by astrocytes (Figure 3B,D). Astrocyte NO production induced by chemical hypoxia (cyanide, myxothiazol) was unaffected by L-NAME (Figure S3A,B). Inhibition of mitochondrial respiration with rotenone (2 µM) or cyanide (100 µM) had no effect on NO production in cultured cerebellar and cortical neurons (Figure S4).

Mitochondrial inhibition with rotenone was previously reported to selectively occlude the hypoxia-induced responses of the prototypical mammalian oxygen sensors - carotid body glomus cells^24,25^. We next applied rotenone by microinjection in the vicinity of the imaged arterioles *in vivo*. Rotenone had no effect on resting vessel diameter (8.2±1.1 µm *vs* 7.9±0.8 µm in control conditions; n=7 in each group; p=0.80), but completely blocked the dilations of cortical arterioles induced by hypoxia (Figure 3E,F).

**Figure 4:**
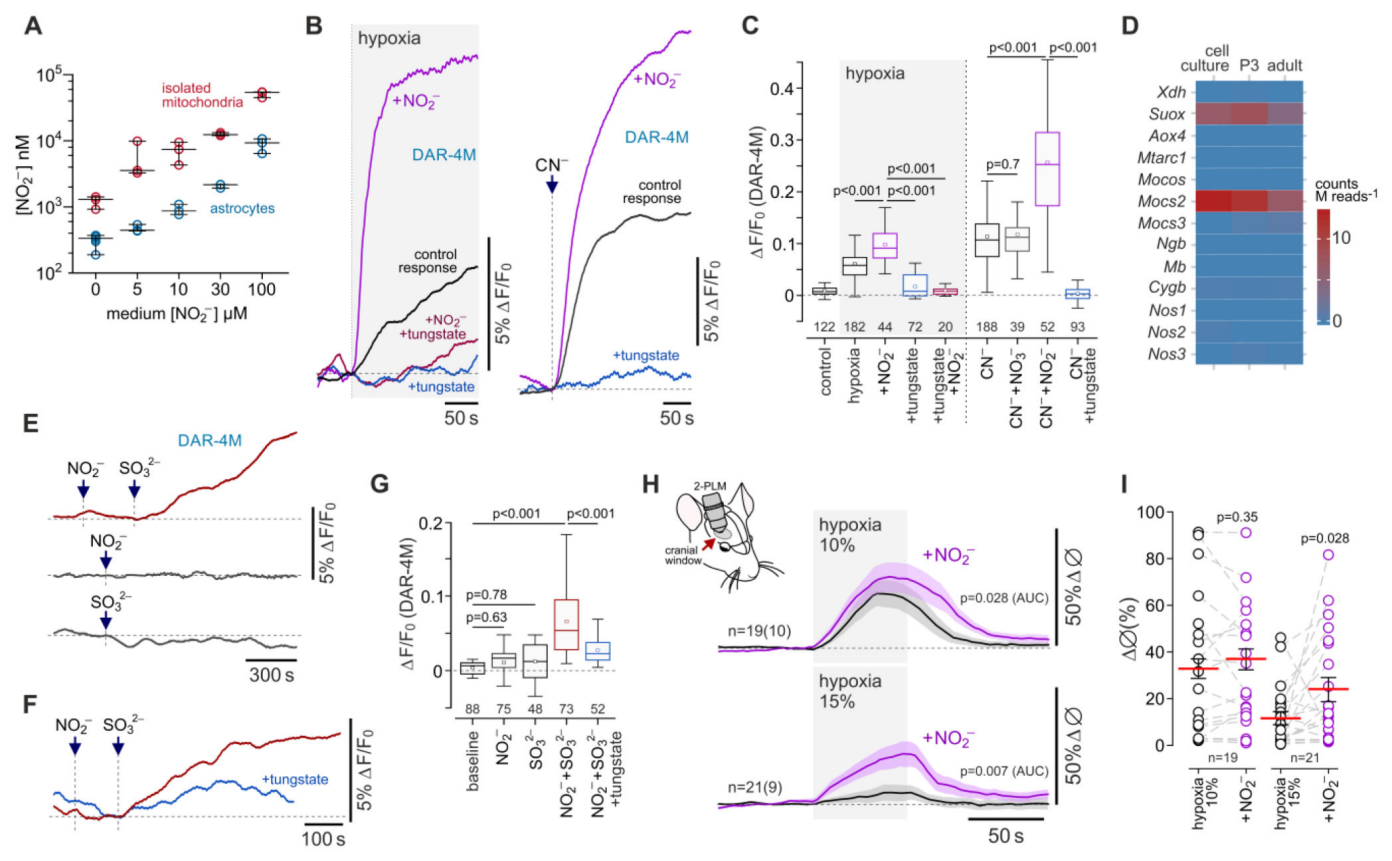
During hypoxia astrocytes produce nitric oxide via nitrite reduction. (A) Astrocyte mitochondria accumulate nitrite. Summary data illustrating measurements of nitrite concentration ([NO_2_^-^]) in astrocytes and isolated astrocyte mitochondria that were incubated for 1 h with NaNO_2_ (0-100 µM) in aerobic conditions. (B) Astrocyte NO production induced by hypoxia or inhibition of complex IV with cyanide (100 µM) is augmented by supplemental NO_2_^-^ (100 µM) and is abolished in cells incubated with tungstate (0.5 mM), which replaces molybdenum with tungsten in molybdopterin-containing enzymes. (C) Summary data illustrating peak changes in DAR-4M fluorescence (reporting NO production) recorded in cortical astrocytes in response to hypoxia or mitochondrial inhibition with cyanide in the absence or presence of supplemental NO_2_^-^, nitrate (NO_3_^-^), and after the incubation with tungstate. *p* values, ANOVA. (D) RNAseq data illustrating the expression of genes encoding metalloproteins with nitrite reductase activity and other relevant proteins in cultured cortical astrocytes, and acutely isolated cortical astrocytes of neonatal (P3) and young adult rats. *Xdh*, xanthine dehydrogenase/oxidase; *Suox*, sulfite oxidase; *Aox4*, aldehyde oxidase 4; *Mtarc1*, mitochondrial amidoxime reducing component; *Mocos*, molybdenum cofactor sulfurase; *Mocs2* and *3*, molybdenum cofactor synthesis genes; *Ngb*, neuroglobin; *Mb*, myoglobin; *Cygb*, cytoglobin; *Nos1*, neuronal NOS; *Nos2*, inducible NOS; *Nos3*, endothelial NOS. (E,F) Sulfite (SO_3_^2-^; 0.5 mM) triggers NO production in astrocytes in the presence of NO_2_^-^ (100 µM). (G) Summary data illustrating peak changes in DAR-4M fluorescence recorded in cortical astrocytes in response to NO_2_^-^, SO_3_^2-^, SO_3_^2-^ in the presence of NO_2_^-^, and in response to SO_3_^2-^ plus NO_2_^-^ in cells incubated with tungstate. *p* values, Kruskal-Wallis ANOVA. In C and G, numbers above the horizontal line indicate the numbers of individual astrocytes recorded in 4-8 separate cultures prepared from 3-6 animals. (H) Hypoxia-induced vascular dilations in the cerebral cortex are augmented following systemic treatment with NaNO_2_ (1 mg kg^-1^, i.v.). Shown are overlaid profiles of changes in the diameter of penetrating cortical arterioles in response to two sequential episodes of hypoxia (10% or 15% inspired O_2_) before and after the administration of nitrite. (I) Summary data illustrating peak hypoxia-induced increases in cortical arteriole diameter in control conditions and after nitrite administration. The data are shown as individual values and means ± SEM. *p* values, Wald test on linear mixed effect model with random intercepts.

Measurements of [NO_2_^-^] in astrocytes or isolated astrocyte mitochondria following incubation of cells with NO_2_^-^ (0, 5, 10, 30, 100 µM) showed that in aerobic conditions astrocyte mitochondria accumulate NO_2_^-^ (Figure 4A). Under resting conditions, the intracellular concentration of NO_2_^-^ in astrocytes was measured at 0.28±0.04 µM, and the concentration of NO_2_^-^ in astrocyte mitochondria was measured at 1.3±0.2 µM. [NO_2_^-^] increased exponentially with a rising concentration of NO_2_^-^ in the medium, reaching 8.8±0.9 µM (p=0.020) in astrocytes (intracellular) and 52.8±3.2 µM (p=0.004) in astrocyte mitochondria in the presence of 100 µM extracellular NO_2_^-^ (Figure 4A). In the presence of supplemental NO_2_^-^ (100 µM) (but not nitrate, NO_3_^-^), NO production by astrocytes in response to hypoxic or chemical hypoxia (cyanide) was greatly enhanced (Figure 4B,C). This effect was blocked by inhibition of anion transport with 4,4’-Diisothiocyano-2,2’-stilbenedisulfonic acid (100 µM) (Figure S3C), pointing to the existence of mechanism(s) that transport nitrite across cell membranes.

Several metalloproteins can facilitate generation of NO by transferring electrons for nitrite reduction^18,19^. To identify the astroglial mechanism responsible for nitrite reduction in hypoxic conditions, we analysed RNAseq data to evaluate the expression of haem-containing and molybdenum (Mo)-bound molybdopterin cofactor proteins and related enzymes in cultured astrocytes, as well as in astrocytes acutely isolated from the cerebral cortex of neonatal (P3) and adult rats (Figure 4D). High relative expression of genes encoding molybdenum cofactor-containing mitochondrial enzyme sulfite oxidase (*Suox* gene) and molybdenum cofactor synthesis protein (*Mocs2* gene; involved in the synthesis of molybdopterin cofactor, required for biochemical activation of molybdenum) was detected in cultured and acutely isolated cortical astrocytes (Figure 4D). High expression of *Suox* and *Mocs2* in cortical astrocytes was confirmed by analysis of data from a publicly available mouse brain transcriptome database^26^ (Figure S5).

If tungstate is added to the medium, tungsten effectively replaces molybdenum in molybdopterin cofactor-containing enzymes^27^. We found that after 24 h of incubation with sodium tungstate (0.5 mM), cultured astrocytes were no longer able to produce NO in response to hypoxic or chemical hypoxia (Figure 4B,C), indicating that a molybdopterin cofactor protein is responsible for nitrite reduction in these conditions. Xanthine oxidase and aldehyde oxidase (both cytosolic proteins) did not appear to be involved, as two potent inhibitors of these enzymes, allopurinol (50 µM) and oxypurinol (20 µM)^18^, had no effect on hypoxia-induced NO production by astrocytes (Figure S3D). Sulfite oxidase (which is highly expressed in cortical astrocytes; Figure 4D), catalyzes the oxidation of sulfite (SO_3_^2-^) in the intermembrane space of mitochondria^28^. We next hypothesized that if sulfite oxidase is responsible for nitrite reduction in astrocytes, then substrate (sulfite) supplementation should trigger NO production in these cells. Indeed, in the presence of 100 µM NO_2_^-^, the addition of sulfite (SO_3_^2-^; 0.5 mM) evoked NO production by astrocytes in aerobic conditions (Figure 4E,G). Neither NO_2_^-^ (100 µM) nor SO_3_^2-^ (0.5 mM) facilitated NO production when applied individually (Figure 4E,G). NO production induced by SO_3_^2-^ in the presence of NO_2_^-^ was reduced in astrocytes that were incubated with tungstate (Figure 4F,G). These data suggested that sulfite oxidase can effectively transfer electrons for nitrite reduction in astrocyte mitochondria.

If the identified mechanism of NO production contributes to hypoxia-induced increases in cerebrovascular flow, then supplemental NO_2_^-^ would be expected to augment the dilations of cerebral vasculature in response to decreases in inspired O_2_. Systemic administration of nitrite (1 mg kg^-1^) had no effect on the peak amplitude of cortical arteriole dilations induced by 10% inspired O_2_ but increased the overall response (0.12±0.003 AUC *vs* 0.11±0.002 AUC in control conditions; 19 vessels recorded in 10 animals; p=0.028; Figure 4H,I). This effect of nitrite was relatively small and could potentially be explained by 10% inspired O_2_ causing near-maximal arteriole dilations and exhaustion of the cerebrovascular reserve. Therefore, we next determined the effect of supplemental NO_2_^-^ on cerebrovascular responses to moderate hypoxia (Figure 4H,I). Peak dilations of cortical arterioles induced by 15% inspired O_2_ were increased by 100% following systemic administration of NO_2_^-^ (diameter increase by 24±5% *vs* 12±3% in control conditions; 21 vessels recorded in 9 animals; p=0.028; Figure 4H,I). Nitrite administration had no effect on Ca^2+^ responses in astrocyte cell bodies and perivascular endfeet induced by 10% or 15% inspired O_2_ (Figure S1B).

**Figure 5:**
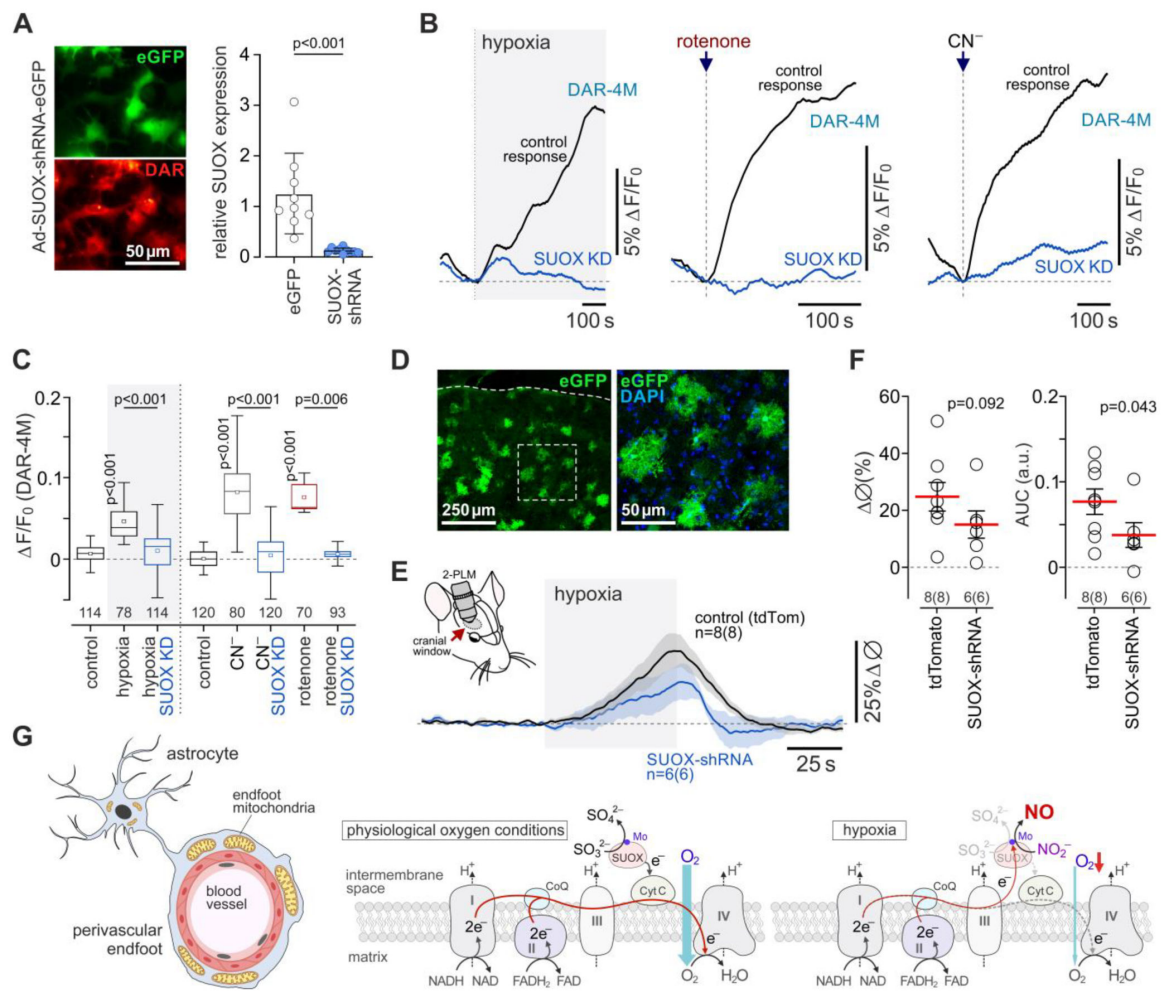
During hypoxia nitric oxide is produced by the enzyme sulfite oxidase in astrocyte mitochondria. (A) Astrocytes in culture transduced to express sulfite oxidase shRNA and loaded with DAR-4M. SUOX-shRNA reduced the expression of sulfite oxidase in astrocytes by 90%. (B) Knockdown of sulfite oxidase (SUOX-KD) blocks NO production in astrocytes induced by hypoxia or mitochondrial inhibition with rotenone (2 µM) or cyanide (100 µM). Each trace illustrates averaged changes in DAR-4M fluorescence recorded in 7-20 individual astrocytes in separate experiments. (C) Summary data illustrating peak changes in DAR-4M fluorescence in response to hypoxia or mitochondrial inhibition with rotenone or cyanide recorded in cortical astrocytes transduced to express eGFP or SUOX-shRNA (SUOX-KD). Numbers above the horizontal line indicate the numbers of individual astrocytes recorded in 4-8 separate cultures prepared from 3-6 animals. *p* values, ANOVA. (D) Representative example of SUOX-shRNA expression in the cerebral cortex of an adult mouse that received an intracerebroventricular administration of AAV5-U6-SUOX-shRNA-eGFP vector at P1. (E) Hypoxia-induced cerebrovascular responses are reduced by SUOX-shRNA expression. Shown are changes in the diameter of penetrating cortical arterioles in response to 10% inspired O_2_. (F) Summary data illustrating peak and integral hypoxia-induced changes in cortical arteriole diameter in mice transduced to express tdTomato or SUOX-shRNA. The data are shown as individual values and means ± SEM. *n=* number of vessels (number of animals). *p* values, unpaired *t*-test. (G) Schematic drawing of the mitochondrial ETC illustrating the proposed mechanism of NO production by astrocytes in response to hypoxia. In aerobic (physiological oxygen level) conditions sulfite oxidase catalyzes the oxidation of sulfite to sulfate and transfers electrons to cytochrome *c*. During hypoxia, when mitochondrial respiration is inhibited, and complex III and cytochrome *c* are fully reduced, sulfite oxidase transfers electrons and facilitates proton donation to reduce nitrite to NO.

To test the hypothesis that sulfite oxidase is responsible for hypoxia-induced NO production by astrocytes and dilations of cerebral blood vessels, we next used viral vectors to express short hairpin RNA (SUOX-shRNA) to knock down the expression of this enzyme in cultured astrocytes and *in vivo*. It was found that SUOX-shRNA reduced the expression of sulfite oxidase in astrocytes by 90% (Figure 5A) and blocked the astrocyte NO production induced in response to hypoxia or mitochondrial inhibition with rotenone or cyanide (Figure 5B,C). Intracerebroventricular injections of AAV5-U6-SUOX-shRNA-eGFP vector in neonatal mice established a patchy expression of the transgene in cortical astrocytes of 3-4 months old mice (Figure 5D). SUOX-shRNA expression in the cortex had no effect on resting vessel diameter (7.3±0.7 µm, n=6 *vs* 7.9±0.7 µm in controls, n=8; p=0.58) and peak dilations of cortical arterioles, but reduced the overall vasodilatory responses induced by 10% inspired O_2_ by 51% (7.7±0.2 AUC *vs* 3.8±0.1 AUC in controls; p=0.043; Figure 5E,F).

## Discussion

The data obtained in this study suggest that astrocytes respond to hypoxia by producing NO via nitrite reduction in mitochondria to dilate intraparenchymal cerebral blood vessels and increase brain tissue perfusion. The fact that the effect of chemical inhibition of mitochondrial respiration mimics, but also occludes the effect of hypoxia, points to mitochondria as the site of NOS-independent NO production in astrocytes. Blockade of NO production by tungstate indicates that a molybdenum-molybdopterin containing enzyme is responsible for hypoxia-induced nitrite reduction in astrocytes. Mammals express only four members of this family of metalloproteins^18,19^ and our transcriptome analysis revealed high relative expression of sulfite oxidase in cortical astrocytes. In aerobic conditions and in the presence of supplemental nitrite, astrocytes generated NO in response to sulfite (SO_3_^2-^). Knockdown of sulfite oxidase expression in astrocytes prevented the effect of hypoxia on NO production in these cells and reduced the cerebrovascular response to systemic hypoxia. Collectively, these lines of evidence suggest that during hypoxia sulfite oxidase is responsible for NO production by astrocyte mitochondria (Figure 5G).

In contrast to NO synthesis by enzymes of the NOS family, the generation of NO by this mechanism does not require molecular oxygen. This would be particularly advantageous in conditions of reduced oxygen supply and/or increased oxygen demand when rapid NO release and signalling can effectively increase brain tissue perfusion. Low pH favours the reduction of nitrite to NO by metalloproteins, which transfer an electron and facilitate proton donation^18,19^. Sulfite oxidase is structurally similar to plant nitrate reductase^29^, and is located in proximity to cytochrome *c* within the mitochondrial intermembrane space^30^ where conditions for nitrite reduction appear to be ideal (Figure 5G), especially in astrocytes as these cells have a loosely assembled mitochondrial respiratory chain and a propensity for electron leak^21^. We hypothesise that electrons for nitrite reduction at the molybdenum centre of sulfite oxidase are derived from complex III (Figure 5G).

There is evidence of significant gradients in brain tissue oxygen concentration, even at normal arterial PO_2_ and saturation^7,8^, suggesting that some brain regions may experience critical reductions of oxygen supply^9,10^. Our data point to an important physiological role of astrocyte mitochondria, densely packed in the perivascular endfeet^31^, which cover all penetrating and intraparenchymal cerebral blood vessels (Figure 5G). The data suggest that astrocyte mitochondria function as both the physiological *sensors* of brain tissue oxygen and the *effectors* that can rapidly dilate the associated cerebral blood vessels via release of NO. We hypothesise that this mechanism of NO production by astrocyte mitochondria dynamically matches regional cerebral perfusion to brain tissue oxygen demand and contributes to the increases in global brain blood flow during systemic hypoxia.

### Limitations of the Study

This study contributes to our understanding of the mechanisms underlying oxygen sensing in the brain. The data show that in response to hypoxia the mitochondrial enzyme sulfite oxidase generates NO via reduction of nitrite. The lack of pharmacological tools to manipulate sulfite oxidase activity limited our ability to analyse its function in detail. The use of viral vectors to drive the expression of sulfite oxidase shRNA in astrocytes was highly effective in knocking down the expression of the enzyme (by 90%), but shRNA took several days to achieve an effect that was also irreversible. In this study we used both female and male animals without differentiation. Although we did not notice any obvious differences in the responses and observed effects between individual replicates, the lack of formal analysis of sex-related differences in the described mechanism is a limitation of the study. Another limitation is the use of anaesthesia in the *in vivo* experiments, and future studies involving measurements of hypoxia-induced changes in cerebral blood flow could be performed in awake animals. Finally, in this study our main objective was to study the cellular and physiological responses to acute hypoxia. The role of NO produced by sulfite oxidase in astrocyte mitochondria in the mechanisms underlying the responses and adaptation of the brain to chronic hypoxia remains to be investigated.

## STAR★Methods

### Key resources table

**Table:** 

REAGENT or RESOURCE	SOURCE	IDENTIFIER
Antibodies		
Chicken anti-green fluorescent protein (GFP)	Abcam	AB13970
Donkey anti-chicken Alexa Fluor 488	ThermoFisher	A78948
Anti-GLAST (ACSA-1) phycoerythrin antibody	Miltenyi Biotec	130-118-483
Bacterial and virus strains		
AAV5-U6-SUOX-shRNA-eGFP	Vector Biolabs	shAAV-290554
AAV5-GFAP-tdTomato	Vector Builder	N/A
AAV5-CMV-geNOp	Next Generation Fluorescence Imaging	N/A
Ad-CMV-eGFP	Vector Builder	N/A
Ad-SUOX-shRNA-eGFP	Vector Biolabs	ADV-290554
Chemicals, peptides, and recombinant proteins		
Dextran, Texas Red^™^, 70,000 MW, Neutral	ThermoFisher	D1830
Fluorescein isothiocyanate–dextran	Sigma	FD40
Oregon Green^™^ 488 BAPTA-1, AM, cell permeant	Invitrogen	O6807
DAR-4M	Sigma	D9194
N(ω)-nitro-L-arginine methyl ester	Cayman Chemical	80210
Rotenone	Sigma	R8875
Myxothiazol	Sigma	T5580
KCN	Sigma	60178
FCCP	Sigma	C2920
Oligomycin	Sigma	04876
Tungstate	Sigma	223336
Sodium azide	Sigma	S2002
NaNO_2_	Sigma	237213
Anti-GLAST (ACSA-1) MicroBead Kit	Miltenyi Biotec	130-095-825
Experimental models: Cell lines		
Primary cortical astrocytes	N/A	N/A
Experimental models: Organisms/strains		
Sprague-Dawley rats	Charles River UK	N/A
C57BL/6J	Charles River UK	N/A
CD1 IGS	Charles River UK	N/A
Oligonucleotides		
qPCR primer Suox: Rn00579670_g1	ThermoFisher	4331182
Software and algorithms		
Andor iQ3 software	Andor	N/A
Spike2 7.12	Cambridge Electronics Design	https://ced.co.uk/products/spike2
Origin Pro	OriginLab	https://www.originlab.com/origin
FV10 ASW	Olympus	https://www.olympus-lifescience.com/en/

### Resource availability

#### Lead contact

Further information and requests for resources and reagents should be directed to and will be fulfilled by the lead contact, Alexander V. Gourine (a.gourine@ucl.ac.uk).

### Materials availability

This study did not generate new unique reagents.

### Experimental models and subject details

All animal experiments were performed in accordance with the European Commission Directive 2010/63/EU (European Convention for the Protection of Vertebrate Animals used for Experimental and Other Scientific Purposes) and the UK Animals (Scientific Procedures) Act (1986) with project approval from the Institutional Animal Care and Use Committee of the University College London. The animals were group-housed and maintained on a 12-h light/dark cycle (lights on 07:00) and had *ad libitum* access to water and food.

Young Sprague-Dawley rats (100–150 g), adult C57BL/6J mice (2-3 months old), and adult CD1 IGS mice (2-3 months old) of both sexes were used to record hypoxia-induced cortical vascular responses and Ca^2+^ signals in cortical astrocytes. Primary cultures of cortical astrocytes and organotypic slices were prepared from the brains of both female and male rat pups (2- to 10-days post-gestation).

### Method details

#### Two-photon imaging *in vivo*

Rats were anesthetized with isoflurane (2-4% in O_2_-enriched air). The femoral artery and vein were cannulated for recording of arterial blood pressure and administration of drugs, respectively. Isoflurane was discontinued after intravenous administration of urethane (1 g kg^−1^) and α-chloralose (50 mg kg^−1^). Adequate anaesthesia was ensured by stability of arterial blood pressure and heart rate, and lack of cardiovascular responses to a paw pinch. The trachea was cannulated, and the animal was mechanically ventilated using a positive pressure ventilator (tidal volume: ~1 ml per 100 g of body weight; frequency: ~60 strokes min^-1^) with oxygen enriched air (~30% O_2_). During imaging, neuromuscular blockade was established using gallamine triethiodide (induction: 50 mg kg^-1^, i.v.; maintenance: 10 mg kg^-1^ h^-1^, i.v.). Arterial *P*O_2_, *P*CO_2_, and pH were measured regularly and kept within physiological ranges by adjusting the tidal volume and the ventilator frequency. Body temperature was maintained at 37.0±0.5°C. Recordings of heart rate and arterial blood pressure were acquired and analyzed using Power1401 interface and Spike2 software (Cambridge Electronic Design).

Mice were anesthetized with a combination of fentanyl (0.05 mg kg^-1^, i.v.), midazolam (5 mg kg^-1^, i.v.) and medetomidine (0.5 mg kg^-1^, i.v.). Room air supplemented with oxygen (~30% O_2_) was supplied through a nose mask, and mice breathed unaided throughout the experiment. Body temperature was maintained at 37.0±0.5°C.

The head of the animal was secured in a stereotaxic frame and a small circular craniotomy (~3 mm^2^) was made in the parietal bone above the somatosensory cortex. Cortical cells were loaded with a Ca^2+^-sensitive dye Oregon Green BAPTA 1 AM (OGB). OGB was first dissolved in DMSO and Pluronic F127 (20%). The solution containing OGB (1 mM) in artificial cerebrospinal fluid (aCSF; 124 mM NaCl, 3 mM KCl, 2 mM CaCl_2_, 26 mM NaHCO_3_, 1.25 mM NaH_2_PO_4_, 1 mM MgSO_4_, 10 mM D-glucose saturated with 95% O_2_/5% CO_2_, pH 7.4) was delivered by microinjection via a glass micropipette at 2-4 sites within the targeted area of the cortex. The exposed surface of the brain was then covered with agarose (1%) and protected with a glass coverslip secured to the skull with a headplate and acrylic dental cement. Intravascular fluorescent dyes Texas Red™ (15 mg kg^-1^; MW 70,000, ThermoFisher) or Fluorescein isothiocyanate (FITC)-Dextran (15 mg kg^-1^; MW 40,000, Sigma) were administered intravenously.

Vascular and cellular responses in the cortex were recorded using an Olympus FV1000 microscope (Olympus), equipped with MaiTai HP DeepSee laser (Spectra-Physics). A 25x water-immersion objective (XLPlan N, NA 1.05; Olympus) was used. Fluorophores were excited in two-photon XYZ-t mode at 800 nm and images were acquired ~100-200 μm deep from the surface of the brain. Cortical arterioles (penetrating and intraparenchymal) were identified anatomically and by fluorescence of Texas Red or FITC-Dextran. Astrocytes were identified by their characteristic anatomical features such as endfeet. Laser power was kept to a minimum to reduce phototoxicity. Time-lapse recordings were made for up to 10 min with a period of hypoxia (10% or 15% O_2_ in the inspired gas, balanced with N_2_) lasting for ~1 min. This protocol was selected in a series of pilot trials as it was found to evoke robust responses of brain parenchymal arterioles reaching peak dilations within this time period.

#### Processing of two-photon imaging data

Rigid-body shifts in the recorded data due to in-plane movements of the brain were corrected using the red fluorescence channel and an average over the first 20 frames as a template. The recorded imaging data were denoised by a two-step truncated singular value decomposition (SVD) projection algorithm. In short, each colour channel video was split into overlapping spatial windows (“patches”, 8x8 pixels), each window was then serialized to form a 2D matrix with single-pixel signals in rows; these matrices were approximated with truncated SVD. Temporal singular vectors were then collected in larger windows and approximated by a second truncated SVD. Inverse SVD transform provided denoised estimates of changes in fluorescence within each patch; estimates from overlapping patches were then averaged.

To measure changes in cortical vessel diameter, a line-scan was manually drawn across the widest part of the vessel and the lumen of the vessel was segmented using Chang-Vese active contours with constrains^32,33^. Cell bodies of astrocytes were identified by an adaptive thresholding algorithm. Regions of interest (ROIs) corresponding to astrocyte cell bodies were manually selected for analysis. Changes in OGB fluorescence within each ROI were used for Ca^2+^ signal detection^33,34^. An increase in OGB fluorescence was labelled as a Ca^2+^ transient if gaussian-smoothed (sigma=1 frame) ΔF/F signal exceeded a threshold of 2.5 standard deviations (obtained via median absolute deviation robust estimator) for at least 3 consecutive frames.

Astrocyte endfeet were identified as OGB-stained structures encircling penetrating arterioles. To analyse Ca^2+^ responses in the endfeet, a special algorithm was applied to trace the deformations of the endfeet associated with changes in vessel diameter. In each frame, the circumference of an endfoot around a vessel was tracked and resampled along the resulting closed path to a constant number of steps (360), corresponding to rotation degree φ in polar coordinates with the origin at the centre of the vessel. First, a ridge-enhancing filter was applied to each frame in a crop around a vessel of interest in the green fluorescence channel. These ridge-enhanced frames were resampled to polar coordinates with the origin at the vessel centre, and the endfoot was segmented as a brightness-weighted path from φ=0 to φ=360 within the graph of connected local maxima locations with constraint on matching radius coordinates for the start and the end of the path and path length. OGB fluorescence signals were then sampled along the circumference of the endfeet in 360 φ providing 1 vector column of OGB fluorescence per each frame, resulting in a 2D representation of Ca^2+^ changes, with time on the X-axis and the rotation angle of the circumference on the Y-axis. This kymograph of changes in OGB fluorescence was used for the detection of Ca^2+^ signals in the endfeet. Increases in OGB fluorescence above 5% ΔF/F and larger than 64 pixels (frames × degrees) were labelled as Ca^2+^ signals in the endfeet. Maximal projections of changes in ΔF/F signals along the Y-axis were used to plot Ca^2+^ signal traces.

#### Intracerebroventricular injections of viral vectors

In a series of validation trials conducted in rats and mice the expression of SUOX-shRNA induced by AAV5-U6-SUOX-shRNA-eGFP vector in the rat brain was found to be very limited. Therefore, mice were used in the experiments involving targeting of cortical astrocytes to express SUOX-shRNA. The viral vectors were administered to 1-day post-gestation (P1) neonatal CD1 IGS mice via unilateral intracerebroventricular injection (AP: -1.5mm; ML: 0.5mm; DV: 1.3mm). The pups were anesthetized with isoflurane and injected with viral vector AAV5-U6-SUOX-shRNA-eGFP (10^11^ viral genomes; Vector Biolabs) or AAV5-GFAP-tdTomato (control; 10^8^ viral genomes, Vector Builder) using a 33-gauge needle (injection volume <1.5 µL). After the injections, the pups were returned to the dam and were used in experiments at 7-8 weeks of age.

#### Cell cultures and organotypic slice cultures

Primary cultures of cortical astrocytes were prepared from the brains of rat pups (P2-3 of either sex)^35–38^. The animals were euthanized by exposure to isoflurane, the brains were removed, and the cortical regions were separated by dissection. After isolation, the cells were plated on poly-D-lysine-coated coverslips and maintained at 37°C in a humidified atmosphere of 5% CO_2_ and 95% air for a minimum of 10 days before experiments. Viral vectors to express the genetically encoded NO sensor geNOp (AAV5-CMV-geNOp, titre 1.3x10^11^; Next Generation Fluorescence Imaging), enhanced green fluorescent protein (Ad-CMV-eGFP, titre 1.0x10^10^; Vector Builder), or SUOX-shRNA-eGFP (Ad-SUOX-shRNA-eGFP, titre 1.5x10^10^; Vector Biolabs) were added to the incubation medium after 5-7 days from the time of cell culture preparation.

Neuronal cultures were prepared from the cerebellum as the expression of neuronal NOS is highest in this part of the brain^39^. Rat pups (P6-8 of either sex) were euthanized by isoflurane, the brains were removed, and the cerebella were separated in ice cold Hank’s Balanced Salt Solution (HBSS) buffer. Cells were dissociated after tissue incubation with TrypLE enzyme (ThermoFisher; 15 min at 37°C) and suspended in Neurobasal medium containing B-27 supplement, 2 mM L-Glutamine, 25 mM K^+^, 100 U ml^-1^ penicillin, and 0.1 mg ml^-1^ streptomycin. Cells were then plated on poly-D-lysine-coated coverslips and maintained at 37°C in a humidified atmosphere of 5% CO_2_ and 95% air for a minimum of 5 days before experiments.

Organotypic cortical slices were prepared from the brains of rat pups (P8-10 of either sex)^13^. The animals were euthanized by isoflurane, the brains were removed and placed in ice-cold HBSS without Ca^2+^, with added 20 mM glucose (total 25.6 mM), 10 mM MgCl_2_, 1 mM HEPES, 1 mM kynurenic acid, 0.005% phenol red, 100 U ml^-1^ penicillin, and 0.1 mg ml^-1^ streptomycin. A sequence of coronal slices (400 µm) was cut at the level of the somatosensory cortex and plated on Millicell-CM organotypic culture membrane inserts (Merck Millipore). Slices were cultured in a medium containing 50% Optimem-1, 25% foetal bovine serum (FBS), 21.5% HBSS; 25 mM glucose, 100 U ml^-1^ penicillin, and 0.1 mg ml^-1^ streptomycin. After 3 days, the plating medium was removed and replaced with Dulbecco's Modified Eagle medium containing 10% FBS, 2 mM L-Glutamine, 100 U ml^-1^ penicillin, and 0.1 mg ml^-1^ streptomycin. The medium was subsequently replaced twice a week. The slices were used in the experiments after 7-10 days of incubation.

#### Optical recordings of NO production in astrocytes

Optical recordings of NO production in cultured astrocytes were performed using an inverted epifluorescence Olympus microscope, equipped with a cooled CCD camera (Clara model; Andor). The cells were loaded with the NO sensitive fluorescent probe DAR-4M-AM (Sigma; 10 µM, 30 min incubation at room temperature) or transduced to express the genetically encoded NO sensor geNOp. Recordings were performed in a custom-made flow-through imaging chamber at ~32°C in aCSF saturated with 95% O_2_ / 5% CO_2_ (pH 7.4). The rate of chamber perfusion with aCSF was 4 ml min^-1^. DAR-4M fluorescence was excited by using a Xenon arc lamp and an Optoscan Monochromator (Cairn Research) at 560/10 and the fluorescence emission was recorded at 590 nm. geNOp fluorescence was excited at 488/10 nm and the fluorescence emission was recorded at 535 nm.

Hypoxic conditions *in vitro* were induced by the displacement of oxygen in the medium by argon. In all experiments in cell cultures and organotypic slices the hypoxic challenge was applied for 5-15 min. A representative profile of PO_2_ changes in the recording chamber during argon displacement is illustrated by Figure 2F. All test drugs were applied ~10 min before the hypoxic challenge. Imaging data were collected and analyzed using Andor iQ3 software (Andor).

#### Measurements of partial pressure of oxygen (PO_2_)

In the experiments in organotypic slices, PO_2_ was recorded using optical fluorescence probes (250 µm tip diameter, OxyLite™ system, Oxford Optronix) placed on the surface of the slice^13^.

#### Astrocyte sample preparation for nitrite measurements

Accumulation of NO_2_^-^ in astrocytes and astrocyte mitochondria was determined by chemiluminescence assay^40^. After 12 days in culture, astrocytes were washed with phosphate-buffered saline (PBS) and incubated for 60 min in HBSS containing NO_2_^-^ (NaNO_2_) at concentration of 0, 5, 10, 30 or 100 µM. Samples were then washed with PBS (3x), treated with trypsin (5 min), and centrifuged (at 240×g, 5 min). The supernatant was removed, and the cell pellets were resuspended in PBS to washout the extracellular NO_2_^-^. This procedure was repeated twice. After the last wash, the cells were resuspended in 10 ml ddH_2_O to induce osmotic lysis and the samples were flash-frozen and stored at -80°C until assayed. In a separate set of the experiments, pure astroglial cultures were incubated for 60 min with NO_2_^-^ (0, 5, 10, 30 or 100 µM). After harvesting by trypsinization, the cells were washed twice in PBS and incubated on ice for 15 min. Mitochondria were isolated by a series of centrifugations at 600×g, 900×g and 12,000×g. The mitochondrial pellet was then resuspended in ddH_2_O to induce osmotic lysis of the organelles; the samples were frozen and stored at –80°C until assayed.

#### Isolation and purification of astrocytes, RNA sequencing

Young adult male rats (~100 g) and rat pups (P3 of either sex) were used to isolate cortical astrocytes^36^. The animals were euthanized by isoflurane inhalation overdose and the brains were isolated. The cortex was dissected and the meninges were removed. The tissue was enzymatically dissociated to make a suspension of individual cells. The samples were passed through a 45 μm Nitex mesh to remove undissociated cell clumps and after addition of myelin removal beads (Miltenyi Biotec), passed through a MACS column (Miltenyi Biotec). The second (positive) magnetic separation was then performed using astrocyte-specific anti-GLAST (ACSA-1) antibodies conjugated to the magnetic beads (Miltenyi Biotec). Cell purity was assessed by using anti-GLAST (ACSA-1) phycoerythrin antibody (Miltenyi Biotec) and flow cytometry (CyanADP Cytometer, Beckman Coulter). FACS analysis confirmed >95% purity of isolated astrocytes. Purified cells were harvested by centrifugation at 2000× g for 5 min. The cell pellet was then used for RNA extraction. Separately, cultured astrocytes were individually collected using patch pipettes (tip ~5 µm) made of borosilicate glass. One biological replicate consisted of 20-25 pooled cells from 3-4 cultures.

Total RNA was isolated using RNeasy Plus Micro Kit (Qiagen) following the manufacturer’s protocol and RNA quality was assessed using the RNA 6000 Pico Kit on a 2100 Bioanalyzer (Agilent Technologies). RNA sequencing, read mapping, and expression level estimation were performed as described^36^. Reads were aligned to the rat reference genome RGSC3.4.64 with TopHat 1.3.3. Cufflinks v1.0.2 was used to assemble and quantify the transcriptome of each sample. A union set of transcripts in all samples was generated with Cuffcompare, and differential expression was assessed with Cuffdiff. Expression level is reported as fragments per kilobase of transcript sequence per million mapped fragments (FPKM) values.

Expression of genes encoding nitric oxide synthases and all known metalloproteins with nitrite reductase activity^18,19^ were compared across the three conditions: cultured cortical astrocytes (1), and cortical astrocytes acutely isolated from neonatal (2) and young adult (2) rats. Genes included in the analysis were as follows: *Aox4*, aldehyde oxidase 4; *Cygb*; cytoglobin, *Mtarc1*; mitochondrial amidoxime reducing component 1, *Mb*; myoglobin, *Mocos*; molybdenum cofactor sulfurase, *Mocs2*; molybdenum cofactor synthesis 2, *Mocs3*; molybdenum cofactor synthesis 3, *Ngb*; neuroglobin, *Nos1*; nitric oxide synthase neuronal, *Nos2*; nitric oxide synthase inducible, *Nos3*; nitric oxide synthase endothelial, *Suox*; sulfite oxidase, *Xdh*; xanthine dehydrogenase/oxidase. Plots were created using the ggplot2 package in R.

#### Quantitative real-time PCR

Quantitative real-time PCR (RT-qPCR) was used to determine the level of sulfite oxidase mRNA expression in cortical astrocytes transduced to express eGFP (n=9) or SUOX-shRNA-eGFP (n=10). Total RNA was extracted, purified (RNeasy micro kit, 74004, Qiagen), and reverse transcribed using the QuantiTect Reverse Transcription Kit (205311, Qiagen). PCR reactions were performed in 20 μl volumes using the TaqMan Universal Master Mix II (4440040, ThermoFisher) with the final volume of 9 μl cDNA, equivalent to 24 ng RNA sample template per reaction. PCR reactions were performed in duplicates using the TaqMan assay (Suox, Rn00579670_g1, 81 bp amplicon length, ThermoFisher) as detection method, and an Agilent Technologies Aria Mx RT-PCR system (Agilent). Relative *Suox* expression values were calculated using the comparative CT method (ΔΔCt) and presented as arbitrary units of expression, normalized to the expression of the Ubiquitin C gene (Rn01789812_g1, 88 bp amplicon length, ThermoFisher).

#### Immunohistochemistry

Mice were terminally anesthetized with isoflurane and transcardially perfused with saline, followed by ice-cold 4% paraformaldehyde solution. The brains were removed, fixed overnight in 4% paraformaldehyde, and sectioned serially using a cryostat. Brain sections (30 μm) were incubated overnight at room temperature with chicken anti-GFP antibodies (1:500 dilution; Abcam), followed by incubation with fluorochrome-conjugated donkey anti-chicken Alexa Fluor 488 secondary antibodies (1:1,000 dilution; ThermoFisher) for 2 hr. Sections were mounted using Fluoroshield antifade mounting medium. Fluorescent images were acquired using a confocal microscope (Leica SP8) with a 25x objective.

### Quantification and statistical analysis

Data were compared by linear mixed-effects model test for nested data with random intercepts, Kruskal-Wallis ANOVA, one way ANOVA with Tukey post-hoc test, or *t* test as appropriate. Linear mixed-effects models were used to analyze the effects of treatments on vascular responses and Ca^2+^ signals in astrocyte cell bodies and endfeet; the presence or absence of the drug was treated as a fixed effect, and intercepts for each animal and structure of interest were treated as random effects. *P* values were determined by post-estimation inference using Wald tests. Linear mixed-effect model analysis was performed in the "statsmodels" library for Python. The other statistical tests were performed using OriginPro software. The data are shown as individual values and means ± SEM or box-and-whisker plots. Details of the statistical tests applied are provided within the figure legends. Differences with a *P* value of less than 0.05 were considered to be significant.

## Supplementary Material

Supplementary material

## Data Availability

Data reported in this paper will be shared by the lead contact upon reasonable request. The computer code used for the analyses of the *in vivo* imaging data is available via the link: https://doi.org/10.5281/zenodo.10080722. Any additional information required to reanalyze the data reported in this paper is available from the lead contact upon reasonable request.

## References

[1] Howarth C, Gleeson P, Attwell D (2012). Updated energy budgets for neural computation in the neocortex and cerebellum. J Cereb Blood Flow Metab.

[2] Buxton RB (2010). Interpreting oxygenation-based neuroimaging signals: the importance and the challenge of understanding brain oxygen metabolism. Front Neuroenergetics.

[3] Bailey DM (2019). Oxygen, evolution and redox signalling in the human brain; quantum in the quotidian. J Physiol.

[4] Gourine AV, Funk GD (2017). On the existence of a central respiratory oxygen sensor. J Appl Physiol (1985).

[5] Willie CK, Tzeng YC, Fisher JA, Ainslie PN (2014). Integrative regulation of human brain blood flow. J Physiol.

[6] Hoiland RL, Bain AR, Rieger MG, Bailey DM, Ainslie PN (2016). Hypoxemia, oxygen content, and the regulation of cerebral blood flow. Am J Physiol Regul Integr Comp Physiol.

[7] Devor A, Sakadzic S, Saisan PA, Yaseen MA, Roussakis E, Srinivasan VJ, Vinogradov SA, Rosen BR, Buxton RB, Dale AM, Boas DA (2011). “Overshoot” of O_2_ is required to maintain baseline tissue oxygenation at locations distal to blood vessels. J Neurosci.

[8] Kasischke KA, Lambert EM, Panepento B, Sun A, Gelbard HA, Burgess RW, Foster TH, Nedergaard M (2011). Two-photon NADH imaging exposes boundaries of oxygen diffusion in cortical vascular supply regions. J Cereb Blood Flow Metab.

[9] Gjedde A (2002). Cerebral blood flow change in arterial hypoxemia is consistent with negligible oxygen tension in brain mitochondria. Neuroimage.

[10] Ndubuizu O, LaManna JC (2007). Brain tissue oxygen concentration measurements. Antioxid Redox Signal.

[11] Gordon GR, Choi HB, Rungta RL, Ellis-Davies GC, MacVicar BA (2008). Brain metabolism dictates the polarity of astrocyte control over arterioles. Nature.

[12] Attwell D, Buchan AM, Charpak S, Lauritzen M, MacVicar BA, Newman EA (2010). Glial and neuronal control of brain blood flow. Nature.

[13] Angelova PR, Kasymov V, Christie I, Sheikhbahaei S, Turovsky E, Marina N, Korsak A, Zwicker J, Teschemacher AG, Ackland GL (2015). Functional oxygen sensitivity of astrocytes. J Neurosci.

[14] Hoiland RL, MacLeod DB, Stacey BS, Caldwell HG, Howe CA, Nowak-Fluck D, Carr JM, Tymko MM, Coombs GB, Patrician A (2023). Hemoglobin and cerebral hypoxic vasodilation in humans: Evidence for nitric oxide-dependent and S-nitrosothiol mediated signal transduction. J Cereb Blood Flow Metab.

[15] Ide K, Worthley M, Anderson T, Poulin MJ (2007). Effects of the nitric oxide synthase inhibitor L-NMMA on cerebrovascular and cardiovascular responses to hypoxia and hypercapnia in humans. J Physiol.

[16] Pelligrino DA, Wang Q, Koenig HM, Albrecht RF (1995). Role of nitric oxide, adenosine, N-methyl-D-aspartate receptors, and neuronal activation in hypoxia-induced pial arteriolar dilation in rats. Brain Res.

[17] Iwamoto J, Yoshinaga M, Yang SP, Krasney E, Krasney J (1992). Methylene blue inhibits hypoxic cerebral vasodilation in awake sheep. J Appl Physiol (1985).

[18] Bender D, Schwarz G (2018). Nitrite-dependent nitric oxide synthesis by molybdenum enzymes. FEBS Lett.

[19] DeMartino AW, Kim-Shapiro DB, Patel RP, Gladwin MT (2019). Nitrite and nitrate chemical biology and signalling. Br J Pharmacol.

[20] Kapil V, Khambata RS, Jones DA, Rathod K, Primus C, Massimo G, Fukuto JM, Ahluwalia A (2020). The Noncanonical Pathway for In Vivo Nitric Oxide Generation: The Nitrate-Nitrite-Nitric Oxide Pathway. Pharmacol Rev.

[21] Lopez-Fabuel I, Le DJ, Logan A, James AM, Bonvento G, Murphy MP, Almeida A, Bolanos JP (2016). Complex I assembly into supercomplexes determines differential mitochondrial ROS production in neurons and astrocytes. Proc Natl Acad Sci USA.

[22] Kojima H, Hirotani M, Nakatsubo N, Kikuchi K, Urano Y, Higuchi T, Hirata Y, Nagano T (2001). Bioimaging of nitric oxide with fluorescent indicators based on the rhodamine chromophore. Anal Chem.

[23] Eroglu E, Gottschalk B, Charoensin S, Blass S, Bischof H, Rost R, Madreiter-Sokolowski CT, Pelzmann B, Bernhart E, Sattler W (2016). Development of novel FP-based probes for live-cell imaging of nitric oxide dynamics. Nat Commun.

[24] Ortega-Saenz P, Pardal R, Garcia-Fernandez M, Lopez-Barneo J (2003). Rotenone selectively occludes sensitivity to hypoxia in rat carotid body glomus cells. J Physiol.

[25] Arias-Mayenco I, Gonzalez-Rodriguez P, Torres-Torrelo H, Gao L, Fernandez-Aguera MC, Bonilla-Henao V, Ortega-Saenz P, Lopez-Barneo J (2018). Acute O_2_ Sensing: Role of Coenzyme QH(2)/Q Ratio and Mitochondrial ROS Compartmentalization. Cell Metab.

[26] Cahoy JD, Emery B, Kaushal A, Foo LC, Zamanian JL, Christopherson KS, Xing Y, Lubischer JL, Krieg PA, Krupenko SA (2008). A transcriptome database for astrocytes, neurons, and oligodendrocytes: a new resource for understanding brain development and function. J Neurosci.

[27] Sparacino-Watkins CE, Tejero J, Sun B, Gauthier MC, Thomas J, Ragireddy V, Merchant BA, Wang J, Azarov I, Basu P, Gladwin MT (2014). Nitrite reductase and nitric-oxide synthase activity of the mitochondrial molybdopterin enzymes mARC1 and mARC2. J Biol Chem.

[28] Wang J, Krizowski S, Fischer-Schrader K, Niks D, Tejero J, Sparacino-Watkins C, Wang L, Ragireddy V, Frizzell S, Kelley EE (2015). Sulfite Oxidase Catalyzes Single-Electron Transfer at Molybdenum Domain to Reduce Nitrite to Nitric Oxide. Antioxid Redox Signal.

[29] Hille R, Hall J, Basu P (2014). The mononuclear molybdenum enzymes. Chem Rev.

[30] Klein JM, Schwarz G (2012). Cofactor-dependent maturation of mammalian sulfite oxidase links two mitochondrial import pathways. J Cell Sci.

[31] Göbel J, Engelhardt E, Pelzer P, Sakthivelu V, Jahn HM, Jevtic M, Folz-Donahue K, Kukat C, Schauss A, Frese CK (2020). Mitochondria-Endoplasmic Reticulum Contacts in Reactive Astrocytes Promote Vascular Remodeling. Cell Metab.

[32] Khennouf L, Gesslein B, Brazhe A, Octeau JC, Kutuzov N, Khakh BS, Lauritzen M (2018). Active role of capillary pericytes during stimulation-induced activity and spreading depolarization. Brain.

[33] Marina N, Christie IN, Korsak A, Doronin M, Brazhe A, Hosford PS, Wells JA, Sheikhbahaei S, Humoud I, Paton JFR (2020). Astrocytes monitor cerebral perfusion and control systemic circulation to maintain brain blood flow. Nat Commun.

[34] Fordsmann JC, Murmu RP, Cai C, Brazhe A, Thomsen KJ, Zambach SA, Lonstrup M, Lind BL, Lauritzen M (2019). Spontaneous astrocytic Ca^2+^ activity abounds in electrically suppressed ischemic penumbra of aged mice. Glia.

[35] Gourine AV, Kasymov V, Marina N, Tang F, Figueiredo MF, Lane S, Teschemacher AG, Spyer KM, Deisseroth K, Kasparov S (2010). Astrocytes control breathing through pH-dependent release of ATP. Science.

[36] Turovsky E, Theparambil SM, Kasymov V, Deitmer JW, Del Arroyo AG, Ackland GL, Corneveaux JJ, Allen AN, Huentelman MJ, Kasparov S (2016). Mechanisms of CO_2_/H^+^ sensitivity of astrocytes. J Neurosci.

[37] Theparambil SM, Hosford PS, Ruminot I, Kopach O, Reynolds JR, Sandoval PY, Rusakov DA, Barros LF, Gourine AV (2020). Astrocytes regulate brain extracellular pH via a neuronal activity-dependent bicarbonate shuttle. Nat Commun.

[38] Turovsky EA, Braga A, Yu Y, Esteras N, Korsak A, Theparambil SM, Hadjihambi A, Hosford PS, Teschemacher AG, Marina N (2020). Mechanosensory signaling in astrocytes. J Neurosci.

[39] Bredt DS, Hwang PM, Snyder SH (1990). Localization of nitric oxide synthase indicating a neural role for nitric oxide. Nature.

[40] Hobbs AJ, Fukuto JM, Ignarro LJ (1994). Formation of free nitric oxide from L-arginine by nitric oxide synthase: direct enhancement of generation by superoxide dismutase. Proc Natl Acad Sci USA.

