# Supplementary material for: Astrocytes produce nitric oxide via nitrite reduction in mitochondria to regulate cerebral blood flow during brain hypoxia
Source: Cell Rep. Author manuscript; Available in PMC 2024 Mar 18. (PMC7615749; doi:10.1016/j.celrep.2023.113514)
Supplement: Supplementary material [file EMS194590-supplement-Supplementary_material.pdf]

Cell Reports, Volume *42*

Supplemental information

Astrocytes produce nitric oxide via nitrite reduction in mitochondria to regulate cerebral blood flow during brain hypoxia

Isabel N. Christie, Shefeeq M. Theparambil, Alice Braga, Maxim Doronin, Patrick S. Hosford, Alexey Brazhe, Alexander Mascarenhas, Shereen Nizari, Anna Hadjihambi, Jack A. Wells, Adrian Hobbs, Alexey Semyanov, Andrey Y. Abramov, Plamena R. Angelova, and Alexander V. Gourine

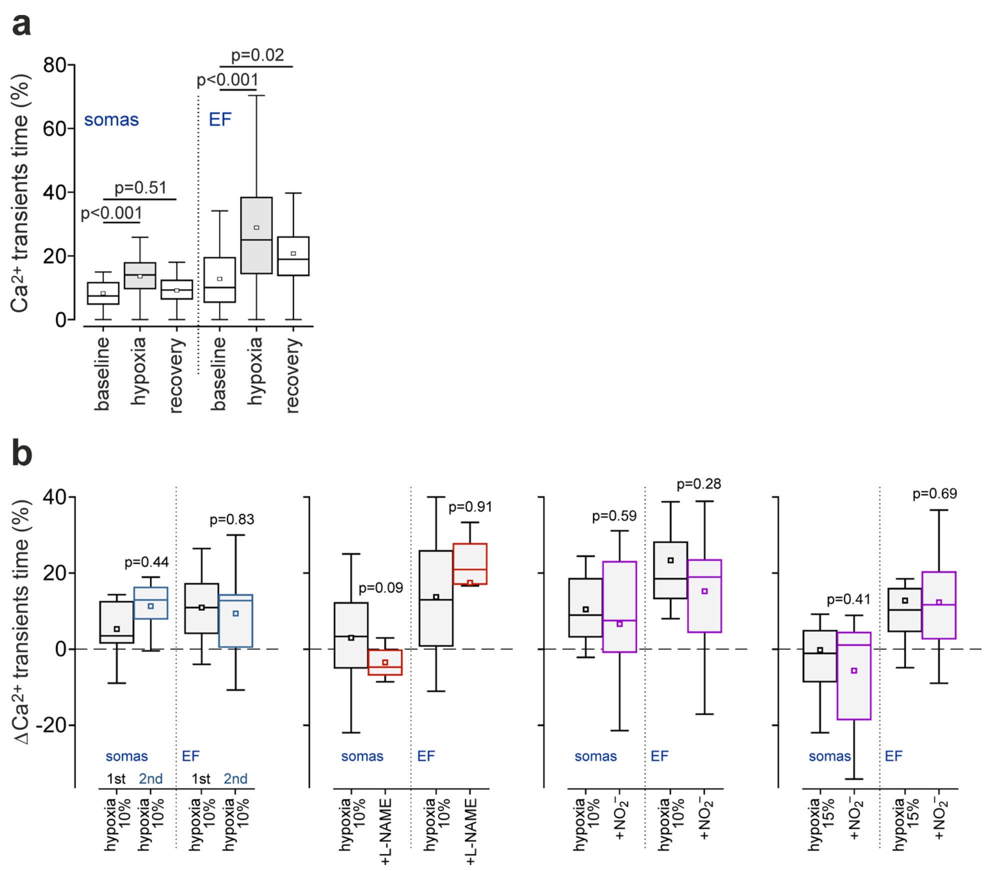

Figure S1: Related to Figure 1 and Figure 4. (**a**) Summary data illustrating the effect of hypoxia on Ca^{2+} transients time (% of time when $[Ca^{2+}]_i$ was elevated) recorded in cell bodies and end-feet of cortical astrocytes in anaesthetised rats. (**b**) Summary data illustrating peak Ca^{2+} responses recorded in cell bodies and end-feet of cortical astrocytes induced by repeated episodes of hypoxia (10% or 15% of O_2 in the inspired gas mixture) in control conditions, following systemic nitric oxide (NO) synthase blockade with N(ω)-nitro-L-arginine methyl ester (L-NAME; 10 mg kg^{-1}, i.v.), or systemic treatment with nitrite (1 mg kg^{-1}, i.v.) in anaesthetised rats. In the box-and-whisker plots the central dot indicates the mean, the central line indicates the median, the box limits indicate the upper and lower quartiles, and the whiskers show the minimum-maximum range of the data. *p* values, Wald test on linear mixed effect model with random intercepts.

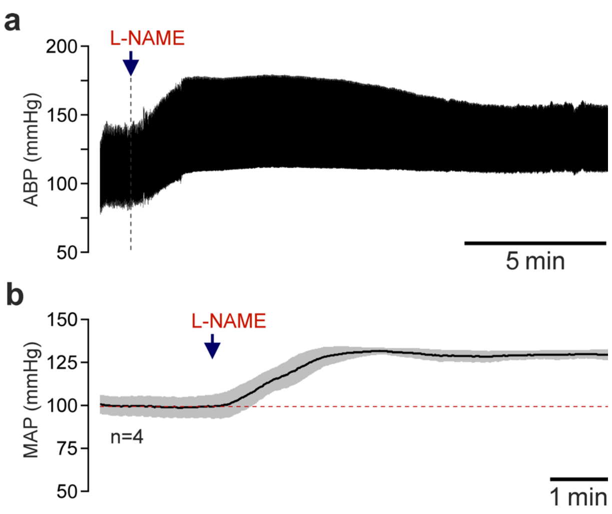

Figure S2: Related to Figure 1. (**a**) A representative recording and (**b**) summary data illustrating changes in arterial blood pressure (ABP) after intravenous administration of N(ω)-nitro-L-arginine methyl ester (L-NAME; 10 mg kg^{-1}) in anaesthetised rats. Sustained elevation of the arterial pressure confirms effective systemic nitric oxide synthase blockade. MAP, mean arterial blood pressure. Data are presented as means ± s.e.m.

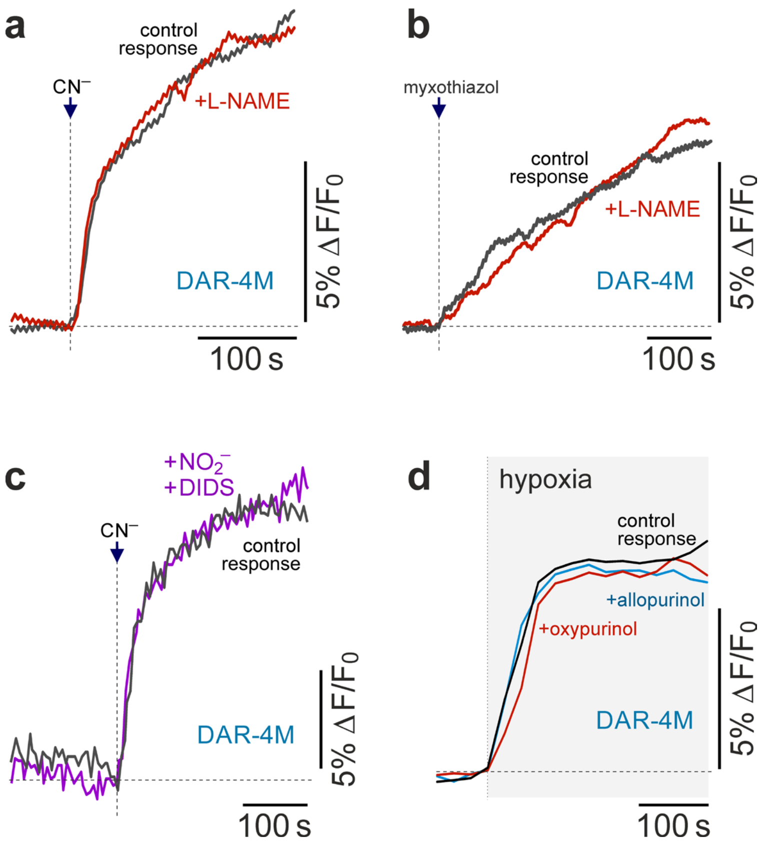

Figure S3: Related to Figure 3 and Figure 4. (**a**) Representative traces showing changes in fluorescence of nitric oxide (NO)-sensitive dye DAR-4M in cultured cortical astrocytes in response to mitochondrial complex IV blockade with KCN (100 μM) in the absence and presence of NO synthase inhibitor N(ω)-nitro-L-arginine methyl ester (L-NAME; 100 μM). (**b**) Representative traces showing changes in DAR-4M fluorescence in astrocytes in response to mitochondrial complex III blockade with myxothiazol (3 μM) in the absence and presence of L-NAME (100 μM). (**c**) Blockade of anion transport with 4,4'-Diisothiocyano-2,2'-stilbenedisulfonic acid (DIDS; 100 μM) prevents the effect of supplemental nitrite (100 μM) on NO production induced by cyanide in astrocytes. (**d**) Representative traces showing hypoxia-induced changes in DAR-4M fluorescence in astrocytes in the absence and presence of xanthine oxidase inhibitors allopurinol (50 μM) or oxypurinol (20 μM).

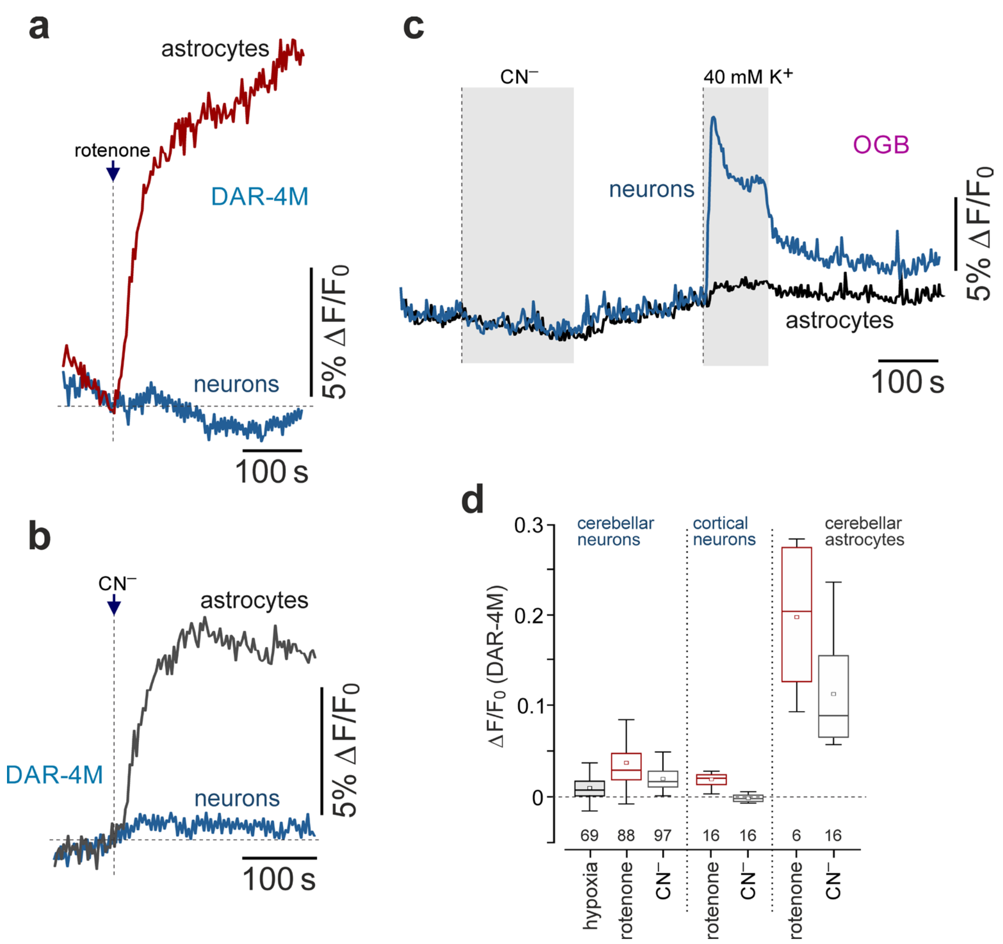

Figure S4: Related to Figure 3. (**a**) Representative traces showing changes in fluorescence of NO-sensitive dye DAR-4M in cerebellar granule neurons and cerebellar astrocytes in response to mitochondrial complex I inhibition with rotenone (2 μM). (**b**) Representative traces showing changes in DAR-4M fluorescence in cerebellar granule neurons and cerebellar astrocytes in response to mitochondrial complex IV blockade with KCN (100 μM). (**c**) Representative traces showing changes in fluorescence of a Ca^{2+}-sensitive dye Oregon Green BAPTA 1 AM (OGB) in cultured cerebellar neurons and cerebellar astrocytes in response to cyanide (100 μM) and KCl (40 mM). Cerebellar granule cells showed robust $[Ca^{2+}]_i$ responses to K^+. (**d**) Summary data illustrating peak changes in DAR-4M fluorescence recorded in cerebellar granule neurons, cortical neurons, and cerebellar astrocytes in response to inhibition of the mitochondrial electron transport chain with rotenone or cyanide.

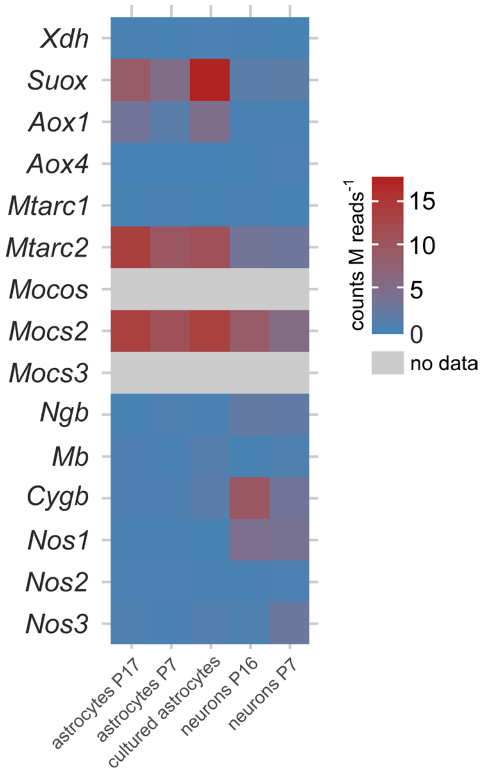

Figure S5: Related to Figure 4. Analysis of RNAseq data from a published astrocyte and neuronal transcriptome database (Cahoy et al.[26]) illustrating the relative expression of genes encoding metalloproteins with nitrite reductase activity and other relevant proteins in astrocytes and neurons acutely isolated from the mouse forebrain as well as in cultured astrocytes. The data show that relative expression of the genes of interest is similar to that obtained in cultured and acutely isolated astrocytes of the rat cerebral cortex (Figure 4d). *Xdh*, xanthine dehydrogenase/oxidase; *Suox*, sulfite oxidase; *Aox1*, aldehyde oxidase 1; *Aox4*, aldehyde oxidase 4; *Mtarc1*, mitochondrial amidoxime reducing component 1; *Mtarc2*, mitochondrial amidoxime reducing component 2; *Mocos*, molybdenum cofactor sulfurase; *Mocs2* and *3*, molybdenum cofactor synthesis genes; *Ngb*, neuroglobin; *Mb*, myoglobin; *Cygb*, cytoglobin; *Nos1*, neuronal nitric oxide synthase (NOS); *Nos2*, inducible NOS; *Nos3*, endothelial NOS.
